# Assessment of setup uncertainty in hypofractionated liver radiation therapy with a breath-hold technique using automatic image registration–based image guidance

**DOI:** 10.1186/s13014-019-1361-6

**Published:** 2019-08-30

**Authors:** Gye Won Choi, Yelin Suh, Prajnan Das, Joseph Herman, Emma Holliday, Eugene Koay, Albert C. Koong, Sunil Krishnan, Bruce D. Minsky, Grace L. Smith, Cullen M. Taniguchi, Sam Beddar

**Affiliations:** 1 Department of Radiation Physics, The University of Texas MD Anderson Cancer Center, Houston, TX 77030 USA; 2 Department of Radiation Oncology, The University of Texas MD Anderson Cancer Center, Houston, TX 77030 USA; 3 The University of Texas Graduate School of Biomedical Sciences at Houston, Houston, TX 77030 USA

**Keywords:** Setup uncertainty, IGRT, In-room CT, Liver radiotherapy, PTV margin

## Abstract

**Background:**

Target localization in radiation therapy is affected by numerous sources of uncertainty. Despite measures to minimize the breathing motion, the treatment of hypofractionated liver radiation therapy is further challenged by residual uncertainty coming from involuntary organ motion and daily changes in the shape and location of abdominal organs. To address the residual uncertainty, clinics implement image-guided radiation therapy at varying levels of soft-tissue contrast. This study utilized the treatment records from the patients that have received hypofractionated liver radiation therapy using in-room computed tomography (CT) imaging to assess the setup uncertainty and to estimate the appropriate planning treatment volume (PTV) margins in the absence of in-room CT imaging.

**Methods:**

We collected 917 pre-treatment daily in-room CT images from 69 patients who received hypofractionated radiation therapy to the liver with the inspiration breath-hold technique. For each treatment, the daily CT was initially aligned to the planning CT based on the shape of the liver automatically using a CT-CT alignment software. After the initial alignment, manual shift corrections were determined by visual inspection of the two images, and the corrections were applied to shift the patient to the physician-approved treatment position. Considering the final alignment as the gold-standard setup, systematic and random uncertainties in the automatic alignment were quantified, and the uncertainties were used to calculate the PTV margins.

**Results:**

The median discrepancy between the final and automatic alignment was 1.1 mm (0–24.3 mm), and 38% of treated fractions required manual corrections of ≥3 mm. The systematic uncertainty was 1.5 mm in the anterior-posterior (AP) direction, 1.1 mm in the left-right (LR) direction, and 2.4 mm in the superior-inferior (SI) direction. The random uncertainty was 2.2 mm in the AP, 1.9 mm in the LR, and 2.2 mm in the SI direction. The PTV margins recommended to be used in the absence of in-room CT imaging were 5.3 mm in the AP, 3.5 mm in the LR, and 5.1 mm in the SI direction.

**Conclusions:**

Manual shift correction based on soft-tissue alignment is substantial in the treatment of the abdominal region. In-room CT can reduce PTV margin by up to 5 mm, which may be especially beneficial for dose escalation and normal tissue sparing in hypofractionated liver radiation therapy.

## Background

Radiation therapy of the liver cancer is challenged by the difficulty in target localization due to numerous sources: the anatomy in the abdominal region is easily affected by organ motion and deformation. The organ motion and deformation are caused by breathing, stomach filling and contraction, peristalsis, variable gas content in bowel loops, and cardiac contractions. To minimize the daily anatomical deformation, many clinics request the patient to withhold the intake of food and fluids for a certain duration of time, usually 3 h, before simulation and treatment. To address the problems posed by breathing motion, many clinics use at least one or a combination of motion management techniques as discussed in the AAPM Task Group 76 [1]. Liver radiation therapy can be categorized as either respiratory-gated or non-gated treatment without or with motion-limiting techniques such as having the patient perform breath hold (BH) [2–4], using active breathing control (ABC) [5–10] or abdominal compression [11], and real-time tumor tracking [12]. Four-dimensional computed tomography (4D CT) is often utilized as well to assess the motion of the target and the surrounding organs-at-risk (OARs) in the planning process. Despite such measures, the sources of uncertainty are not fully eliminated due to involuntary organ motion and deformation that just cannot be controlled, resulting in interfractional and intrafractional uncertainty in the target localization.

Many studies have investigated methods to reduce liver tumor motion and evaluated the residual uncertainty in liver tumor localization when the measures were implemented. Shimohigashi et al. [13] tracked the position of fiducial markers in the liver in 4D CT and reported that liver tumor motion under abdominal compression reached up to 9.4 mm in the anterior-posterior (AP) direction, 3.3 mm in the left-right (LR) direction, and 14.8 mm in the superior-inferior (SI) direction. The study also reported interfractional changes in liver motion under abdominal compression that reached up to 3.0 mm, 2.4 mm, and 3.6 mm in the AP, LR, and SI direction, respectively. Dawson et al. [6] used radiographic images of an implanted hepatic microcoil to evaluate intrafractional tumor motion in patients treated with ABC and revealed a mean SI motion of 2.3 mm (range 1.2–3.7 mm) relative to the bony anatomy. Interfractional changes in the daily liver position were also reported for patients treated with respiratory gating using the BH technique [11]. These studies highlight the presence of residual uncertainty in target localization, despite efforts to minimize the localization uncertainty.

Respiratory-gated radiation therapy with a BH technique is favored due to the non-invasive nature and the capacity to reduce the irradiated volume. The BH technique essentially “freezes” the anatomy during simulation and treatment delivery, so there is less need for a large treatment margin to encompass the whole excursion of the target with breathing motion. For lesions in the liver, which is often tightly surrounded by OARs, such as the duodenum, stomach, and bowel, the BH technique can provide a nominal benefit. Wunderink et al. [14] showed that with a proper implementation of the BH technique, the tumor motion due to breathing can be reduced to less than 5 mm.

The use of image-guided radiation therapy (IGRT) effectively reduces interfractional and intrafractional uncertainty in target localization. IGRT provides a visualization of the patient anatomy at and throughout treatment fractions, and these images allow correction of any localization errors. The use of IGRT is essential in treatments that involve dose escalation, such as hypofractionated radiation therapy; these treatments aim to deliver a high dose to the target with a sharp dose gradient.

To reduce inter-observer and intra-observer dependence in the final image-guided patient alignment and to increase the efficiency of the patient alignment process, many IGRT software packages provide automatic patient alignment tools. Automatic patient alignment tools allow users to choose a region or structure of interest and calculate the image transformation that results in the best image registration of the selected structures in the daily and planning image sets. Most IGRT softwares allow selection of soft tissue such as the liver or the gross tumor volume (GTV) itself as the registration target. This is because soft tissue information is more relevant in tumor localization than other surrogates, such as the bony anatomy.

Despite the benefits, automatic alignment tools can give less-than-desirable alignment results because they do not fully account for the residual uncertainty previously mentioned. Due to the residual uncertainty such as involuntary organ motion and daily organ filling, the daily image set is never identical to the planning image, and the registration algorithm makes compromises to best match the overall image of the registration target in the two image sets. This means that on a daily basis, structures other than the selected registration target may be in substantially different shapes or locations from those in the plan. In addition to the OARs being out of place, the position of the tumor inside an organ may also change due to deformation in the organ itself.

The current study evaluated the uncertainty in automatic patient alignment achieved by the IGRT alignment tool in patients treated for liver lesions with hypofractionated radiation therapy with the inspiration BH technique and in-room CT-based daily IGRT. Using these data, we also determined the recommended margins to be used when the daily imaging modality does not provide sufficient target and OAR visualization.

## Methods

### Patients and radiation treatment

Sixty-nine patients who received hypofractionated radiation therapy to the liver were studied retrospectively. All patients were simulated and treated under inspiration BH using the Real-time Position Management system (RPM, Varian Medical Systems, Palo Alto, CA) with the visual feedback via goggles [15]. Patients were in the supine position in the customized cradle on the wing board with the head resting on the headrest, the arms above the head holding the T-bar, and the knees resting over the knee wedge. The patients were trained to hold their breath at a comfortable level (moderate BH) so that they can reproduce it easily during treatment delivery. During simulation, 3–6 BH scans were acquired for each patient to confirm consistency of the BH, and internal GTV and clinical target volume encompassing targets on all the BH scans were created to compensate for inter-BH variations. In order to deliver 6–9 field intensity-modulated radiation therapy treatment plans with the prescriptions ranging from 37.5 to 90 Gy in 10 or 15 treatment fractions, 6 to 18 BHs were required depending on the status of the patients.

Prior to each treatment, a daily CT scan was acquired using an in-room CT (CT-on-rails, GE Healthcare, Milwaukee, WI) [16]. First the patient was set up at the linac side to the final iso-center by aligning three external markers with the lasers. Then the treatment couch was rotated by 180 degrees to the CT side and the CT moved over the patient on the rails. After the scan, the treatment couch was rotated back to the linac side and the patient was set up again to the final iso-center by aligning the external markers with the lasers. The treatment couch position at the linac side after the rotations should be the same as that before the rotations. The daily CT was initially aligned with the planning CT based on the shape of the liver automatically using an in-house CT-CT alignment software. The algorithm for this software is intensity-based, with the goodness-of-fit between the planning and daily CT calculated as the mean absolute difference in voxel values [17]. Details of the algorithm and tests and applications of this software are described elsewhere [17–20]. At the first treatment fraction for each patient, the attending physician reviewed the automatic alignment to the liver and made manual shift corrections if needed to either better localize the target or move a critical-dose isodose line away from the OARs. For subsequent fractions, either the physicist or radiation therapist who were present during the first fraction setup and who were trained and experienced with CT-CT alignment made manual shift corrections after the automatic alignment, according to the instruction from the attending physician. If needed, the attending physician is paged to assist or approve the alignment. Using this final alignment information as the gold-standard setup, systematic and random uncertainties in the automatic alignment were quantified.

### Uncertainty and margin analysis

A total of 917 daily in-room CT scans and the final alignment information were collected from the treatment records for the 69 patients used for this study. The automatic alignment results were reproduced by re-aligning the daily and planning CT based on the shape of the liver using the CT-CT alignment software.

The discrepancy between the final and automatic alignment was analyzed in the AP, LR, and SI directions and as the magnitude of their three-dimensional (3D) vector. This was used to calculate the setup uncertainty using the method demonstrated in Remeijer et al. [21] (Eqs. 1–4). The setup uncertainty was quantified as two subsets, systematic (Σ) and random (σ) uncertainty. Setup uncertainty along the AP direction is calculated as follows.

1 $$ N=\sum \limits_{p=1}^P{F}_p $$

where *N* (917) is the total number of treated fractions, *P* (69) is the total number of patients, and *F*_*p*_ is the number of treated fractions for patient *p*. The overall mean of all the discrepancy between the final and automatic alignment for all patients along the AP direction is:

2 $$ M=\frac{1}{N}\sum \limits_{p=1}^P\sum \limits_{f=1}^{F_p}{AP}_{pf} $$

where *AP*_*pf*_ is the discrepancy along the AP direction for patient *p* at fraction *f*. Then, Σ and σ are:

3 $$ \varSigma =\sqrt{\frac{P}{N\left(P-1\right)}\sum \limits_{p=1}^P{F}_p{\left({m}_p-M\right)}^2} $$

4 $$ \sigma =\sqrt{\frac{1}{N-P}\sum \limits_{p=1}^P\left({F}_p-1\right)\ast {\sigma}_p^2} $$

where *m*_*p*_ and *σ*_*p*_ are the mean and standard deviation (SD) of the discrepancy along the AP direction for patient *p*. The Σ and σ were also calculated along the other SI and LR directions, as well as for the 3D vector.

On the basis of the quantified Σ and σ, we generated a margin recommendation based on the following margin recipe from van Herk [22]:

$$ Margin=2.5\ \varSigma +0.7\ \sigma $$

## Results

### Discrepancy between the final and automatic alignment

**Fig. 1:**
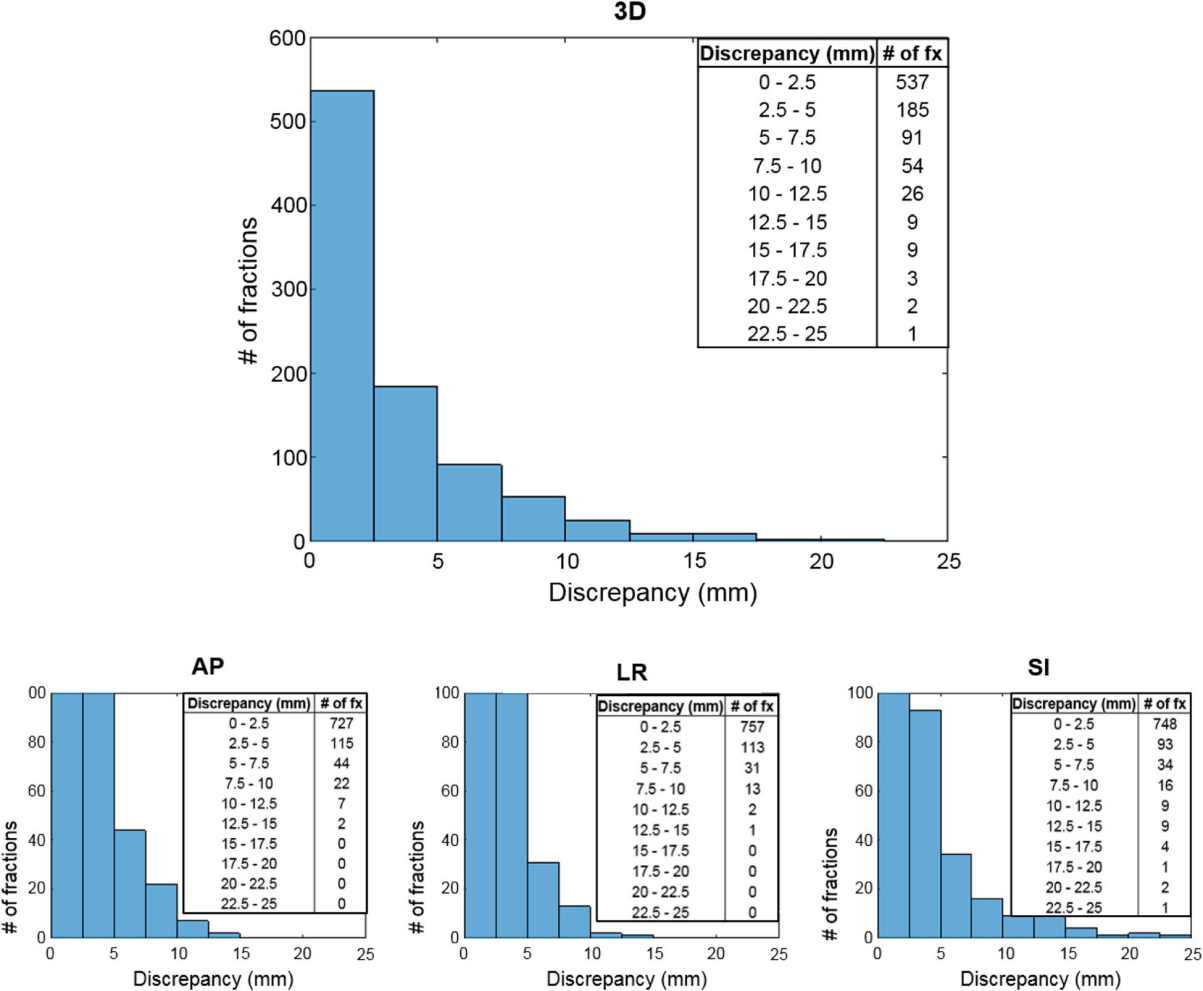
Discrepancy between the final and automatic alignment as the three-dimensional (3D) vector and in the anterior-posterior (AP), left-right (LR), and superior-inferior (SI) directions for 917 treated fractions

The mean and SD of the 3D discrepancy was 2.8 ± 3.8 mm and the median was 1.1 mm. Most fractions had only small discrepancy (Fig. 1). Of 917 fractions, 425 fractions (46.3%) had no discrepancy. This showed that in the treatment of liver lesions under moderate inspiration BH aligning the daily CT with the planning CT based on the shape of the liver was effective in achieving the final alignment.

However, a number of fractions showed finite discrepancy with the maximum 3D discrepancy of 24.3 mm. A total of 344 fractions (37.5%) required a manual shift correction of ≥3 mm, and 50 fractions (5.5%) required a correction of ≥10 mm. The mean and SD of the 3D discrepancy was rather large (2.8 ± 3.8 mm), showing a considerable spread in the extent of the discrepancy between the final and automatic alignment.

**Fig. 2:**
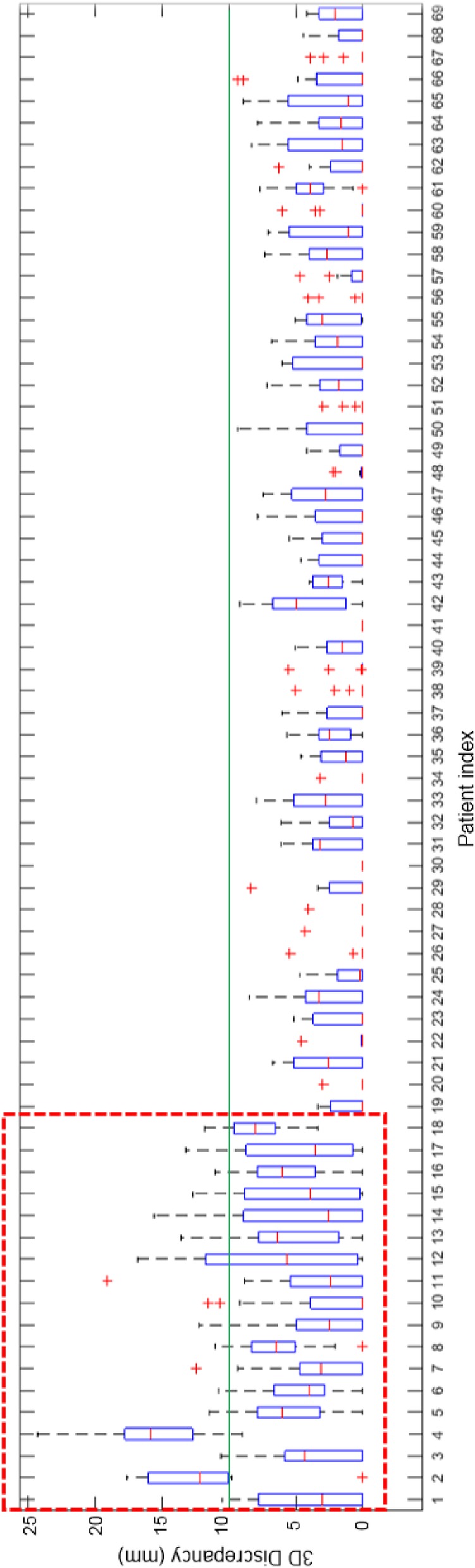
Three-dimensional (3D) discrepancy between the final and automatic alignment for each patient. Patients 1 to 18 (inside the dotted box) had at least one fraction with discrepancy of ≥10 mm

Greater discrepancy was clearly observed along the SI direction than in the other directions (Fig. 1). The maximum discrepancy in the SI direction was 25 mm. The 3D discrepancy for each patient shows that patients who had at least one fraction with a 3D discrepancy of ≥10 mm tended to have greater discrepancy over all fractions in general (Fig. 2).

### Setup uncertainty in the automatic alignment and PTV margins

Table 1 shows the systematic and random uncertainty in the automatic alignment. The overall means of the discrepancy in the AP, LR, and SI directions were 0.3, 0.1, and 0.4 mm, respectively. The Σ ± σ were 1.5 ± 2.2, 1.1 ± 1.9, and 2.4 ± 2.2 mm in the AP, LR, and SI directions, respectively. The PTV margins calculated were 5.3, 4.1, and 7.5 mm in the AP, LR, and SI directions, respectively.

**Table 1:** Overall mean of the discrepancy between the final and automatic alignment, setup uncertainty, and PTV margins calculated

	All patients (69 patients, 917 fractions)			No OAR near target (30 patients, 389 fractions)			OAR near target (39 patients, 528 fractions)		
	AP (mm)	LR (mm)	SI (mm)	AP (mm)	LR (mm)	SI (mm)	AP (mm)	LR (mm)	SI (mm)
Mean, M	−0.3	0.1	0.4	−0.3	0.1	0.3	−0.3	0.3	0.5
Systematic uncertainty, ∑	1.5	1.1	2.4	1.6	0.9	1.5	1.5	1.2	2.9
Random uncertainty, σ	2.2	1.9	2.2	1.9	1.7	2.0	2.4	2.0	2.4
PTV margin	5.3	4.1	7.5	5.3	3.5	5.1	5.3	4.5	8.9

*PTV* planning target volume, *OAR* organ-at-risk, *AP* anterior-posterior, *LR* left-right, *SI* superior-inferior

A further investigation of the final alignment information revealed numerous occasions in which manual shift corrections were made intentionally to shift from the alignment for the best target localization. This was frequently the case when the target was near OARs that were easily deformable (e.g., stomach, duodenum, and small bowel) owing to differences in filling or air content in the organ itself or the surroundings. In such cases, the daily CT was first aligned to the shape of the liver and, if necessary, manual shift corrections were made to best localize the target. If this alignment showed that a critical-dose isodose line was abutting or encompassing the OARs, the daily CT was intentionally shifted out of the alignment to pull the critical-dose isodose line away from the OARs. In these cases, the manual shift corrections were not well-correlated with the uncertainty in target localization, so the uncertainty and margin calculations were performed again without these patients. Thirty-nine patients had one or more OARs that were likely to affect the final alignment. These patients were identified through the visual inspection of the daily CT and final alignment, in addition to the notes from the attending physician in the treatment records.

When the data from the remaining 30 patients (389 fractions) were used to calculate the PTV margins, the margins were 5.3, 3.5, and 5.1 mm in the AP, LR, and SI directions, respectively. The uncertainties and margins calculated for the two groups of patients are also shown in Table 1. Because the manual shift corrections for these patients did not compromise target localization for OAR sparing, this set of margins is more relevant and should be used in treatment plans with high dose conformity and also with the target or the high-dose isodose line not directly abutting the OARs.

## Discussion

Many studies have investigated the uncertainty in targeting liver lesions using various treatment techniques and imaging modalities (Table 2). Dawson et al. [6] used repeated CT scans of patients treated under BH with ABC in treatment setup to evaluate the uncertainty in the location of the liver lesion relative to bony anatomy. Balter et al. [23] reported the random setup uncertainty in patients treated for intrahepatic tumors with the BH technique with ABC and online image guidance through an in-room digital radiography system. Eccles et al. [11] reported interfractional localization uncertainty based on the data from 20 patients who were treated in the liver using the BH technique with ABC. The study used 120 planar AP megavoltage (MV) images from each fraction to quantify the interfractional uncertainty in the SI position of the diaphragm. Hawkins et al. [24] evaluated the residual error in the position of the liver by taking the difference between the initial setup based on orthogonal MV images and the final setup achieved based on cone-beam CT (CBCT). These patients were treated with the exhale BH technique, and the initial setup based on MV images was achieved by aligning the diaphragm in the SI direction and using the vertebral bodies for AP and LR alignment. A study by Case et al. [25] used 314 CBCT scans to evaluate the interfractional uncertainty in the liver position at exhale. The uncertainty was calculated by automatically aligning the vertebral bodies in CBCT and by determining the differences in the daily displacement of the liver. Of 314 CBCT scans, 156 were collected from patients treated with free-breathing with abdominal compression, and the rest were from patients treated without abdominal compression. The systematic and random uncertainties reported in these studies are summarized in Table 2.

**Table 2:** Interfractional uncertainty related to liver target localization from various studies

Study	No. of daily images	No. of patients	Quantity evaluated	AP (mm)	LR (mm)	SI (mm)
Dawson et al. [6]	Not stated	5	Difference in the location of hepatic microcoil relative to bone	M = 3.2 (range 1.2–6.5)	M = 3.3 (range 1.4–5.9)	M = 6.6 (range 2.3–10.9)
Balter et al. [23]	Not stated	8	Random setup uncertainty	σ = 4.1	σ = 4.2	σ = 7.0
Eccles et al. [11]	120	20	Location of diaphragm relative to vertebral body	Not evaluated	Not evaluated	M = 3.4 (range 1.5–7.9)
Hawkins et al. [24]	78 MV images, 72 CBCT images	13	Residual error in liver position after orthogonal MV setup	Σ = 1.3 σ = 3.0	Σ = 1.9 σ = 2.3	Σ = 1.1 σ = 2.7
Case et al. [25]	158	73	Liver position in patients treated with free-breathing technique	M = −1.0 Σ = 1.6 σ = 2.7	M = 1.0 Σ = 1.5 σ = 1.8	M = 1.0 Σ = 3.1 σ = 3.6
	156		Liver position in patients treated with ABC	M = −0.9 Σ = 1.9 σ = 2.2	M = 0.8 Σ = 1.5 σ = 1.8	M = 0.3 Σ = 2.8 σ = 2.6

*AP* anterior-posterior, *LR* left-right, *SI* superior-inferior, *σ*, random uncertainty, *MV* megavoltage, *CBCT* cone-beam computed tomography,*Σ* systematic uncertainty, *M* mean of all setup offsets, *ABC* active breathing control

Results in the current study show that our institution’s current automatic alignment procedure is as effective as the other setup procedures shown in the previous publications, in terms of systematic and random uncertainties. The larger uncertainty in the AP and SI directions than in the LR direction is also consistent with the results from the previous publications and with the common notion that breathing motion mostly affects the anatomy in the SI direction.

Many studies used 3 mm as the threshold for defining substantial offsets that require a correction to the setup and evaluated the likelihood of such offsets occurring [11, 23, 24]_._ Because of the differences in treatment technique and setup procedure among the studies, it is difficult to make direct comparisons or draw a definite conclusion about the probability of substantial offsets occurring. Eccles et al. [11] reported that in 46% of cases the offset in the location of the diaphragm was greater than 3 mm using the BH technique with ABC. Hawkins et al. [24] also reported that in 24 of 72 CBCTs obtained (33%), the residual error was greater than 5 mm. In our study, 38% of treated fractions showed ≥3 mm discrepancy and 21% showed ≥5 mm discrepancy between the final and automatic alignment.

We observed 3D discrepancy as large as 24.3 mm in the automatic alignment. This discrepancy was essentially caught through visual inspections of the automatic alignment and corrected by applying manual shift corrections. The corrections were determined with a high level of confidence owing to the superior soft-tissue contrast level in the daily in-room CT. In addition, patients who had at least one fraction with large discrepancy between the final and automatic alignment tended to have greater discrepancy over all fractions. Therefore, it is recommended to monitor the setup uncertainty on a per-fraction basis and invest additional attention in determining manual shift corrections when large discrepancy is observed.

As mentioned previously, manual shift corrections were performed to accomplish two purposes. The first was to accurately target the lesion, which was not always in the same position due to the residual uncertainty. Sources of residual uncertainty included the finite BH window used in the RPM system and uncontrollable anatomical changes such as involuntary organ motion and daily organ filling. The second purpose of applying manual shift corrections was to pull the radiosensitive OARs, such as the stomach, duodenum, and small bowel, out of the critical-dose isodose lines. Large manual shift corrections were observed in both cases.

Margins shown in Table 1 account for the residual sources of uncertainty after automatic alignment, so it should be used either when the clinic depends on an automatic patient alignment tool to achieve the final setup, or when patient alignment relies on a process that is similar to how an automatic alignment algorithm works. For example, when a clinic does not have access to an IGRT modality with a high level of soft-tissue contrast such as an in-room CT, the patient setup inevitably has to rely on aligning the shape of the liver or the level of the diaphragm. When the user is unable to directly identify the target in the daily images, using the margins from the current study will ensure that the target is still covered by the intended dose. However, these margins are unnecessary when using an IGRT modality with good soft-tissue contrast. An adequate choice of IGRT modality such as in-room CT can thus reduce the setup margin by 4–5 mm in each direction.

**Fig. 3:**
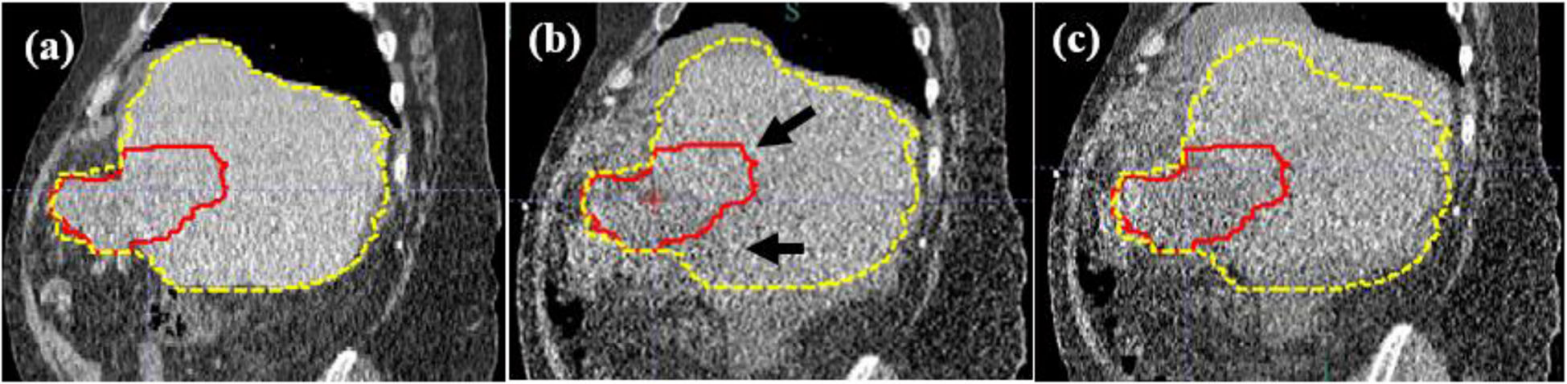
Computed tomography (CT) images showing the maximum manual shift correction of 24.3 mm. **a** Planning CT showing the contours of the liver (dotted yellow) and the gross tumor volume (GTV; solid red). **b** Result of automatic alignment, with the contours of the liver and GTV overlaid on top of the daily CT. The black arrows show the mismatch in the contour and the actual location of the GTV. **c** Result of manual shift correction

For the patient with the maximum 3D discrepancy of 24.3 mm, the daily CT revealed ascites that deformed the shape of the liver (Fig. 3). The automatic alignment tool failed to account for the ascites and registered the liver contours midway in between the ascites and the actual liver (Fig. 3b). The in-room CT clearly showed the presence of ascites and the location of the lesion in the liver, therefore a 24.3 mm manual shift correction was applied to correctly localize the target within the high-dose region (Fig. 3c).

**Fig. 4:**
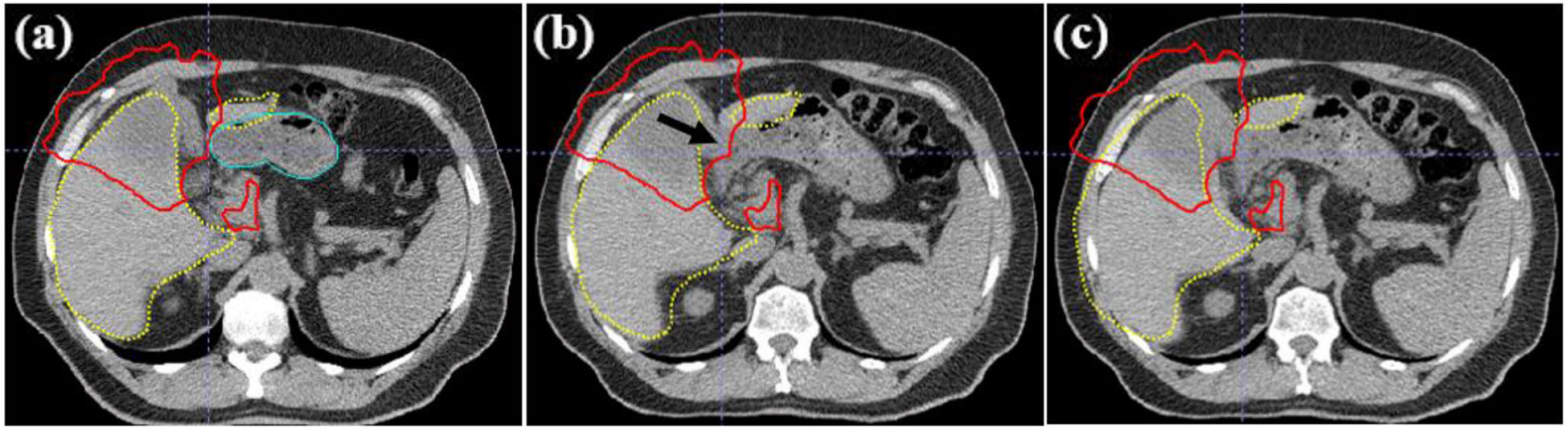
Computed tomography (CT) images showing the left-right manual shift correction of 12.7 mm. **a** Planning CT showing the contours of the liver (dotted yellow) and the 45 Gy isodose line (solid red) abutted the stomach (solid blue). **b** Result of automatic alignment showing a deformation in the shape of the stomach (black arrow). **c** Result of manual shift correction in which the critical dose isodose line was pulled out of the stomach

Images from another patient revealed that large manual shift corrections were applied to shift the OAR out of the high-dose region (Fig. 4). The images from the planning CT (Fig. 4a) and the daily CT (Fig. 4b) showed that the shape of the liver and the location of the lesion had stayed constant from simulation to treatment. However, the stomach had a larger volume than that at the simulation and was falling into the 45 Gy isodose line, which is considered the critical dose for the stomach (Fig. 4b). On the basis of visual inspection of the automatic alignment, a 12.7 mm LR manual shift correction was made to shift the critical dose isodose line away from the stomach (Fig. 4c).

Both cases show that when the appropriate IGRT modality is used, manual shift corrections can be determined to identify and correct for the residual uncertainty. For the current study, in-room CT provided images that were nearly equivalent in quality to the simulation CT and could resolve the details in the abdominal region. The superior soft-tissue contrast allowed physicians to confidently determine manual shift corrections to either chase the target or spare the OARs from the high-dose region. The in-room CT also avoided the problem of limited scan lengths that exists in other IGRT modalities, and this made it easier to treat large-sized patients and patients with large livers.

## Conclusion

The patient setup process using an automatic alignment software aims to provide a setup that is consistent throughout the course of the radiation treatment and is robust to inter-observer and intra-observer variability. The alignment software based on the shape of the liver often fails to account for residual uncertainty and thus requires manual shift corrections following the automatic alignment to achieve the final desired alignment for treatment. The current study quantified the uncertainty in alignment based on the shape of the liver in hypofractionated liver radiation therapy with inspiration BH and automatic image registration-based IGRT. We found that although the automatic alignment was effective in many cases, numerous other cases required manual shift corrections based on soft-tissue alignment. A set of margins was calculated based on the uncertainty in automatic alignment and was recommended to be used when manual shift corrections could not be determined with high confidence. The current study also demonstrated the importance of using the IGRT modality which has a high level of soft-tissue contrast. The use of in-room CT was shown to reduce the PTV margin by 4 to 5 mm and was especially beneficial for dose escalation and normal tissue sparing for hypofractionated liver radiation therapy.

## Data Availability

The datasets used and/or analyzed during the current study are available from the first author on reasonable request.

## Abbreviations

3D: Three-dimensional
4D: Four-dimensional
ABC: Active breathing control
AP: Anterior-posterior
BH: Breath hold
CB: Cone-beam
CT: Computed tomography
GTV: Gross tumor volume
IGRT: Image-guided radiation therapy
LR: Left-right
MV: Megavoltage
OAR: Organ-at-risk
PTV: Planning treatment volume
RPM: Real-time Position Management
SD: Standard deviation
SI: Superior-inferior

## References

[1] Keall P, Mageras GS, Balter JM (2006). The management of respiratory motion in radiation oncology report of AAPM Task Group 76. Med Phys.

[2] Hanley J, Debois MM, Mah D (1999). Deep inspiration breath-hold technique for lung tumors: the potential value of target immobilization and reduced lung density in dose escalation. Int J Radiat Oncol Biol Phys.

[3] Mageras GS, Yorke E (2004). Deep inspiration breath hold and respiratory gating strategies for reducing organ motion in radiation treatment. Semin Radiat Oncol..

[4] Yang W, Fraass BA, Reznik R (2014). Adequacy of inhale/exhale breathhold CT based ITV margins and image-guided registration for free-breathing pancreas and liver SBRT. Radiat Oncol.

[5] Wong JW, Sharpe MB, Jaffray DA (1999). The use of active breathing control (ABC) to reduce margin for breathing motion. Int J Radiat Oncol Biol Phys.

[6] Dawson LA, Brock KK, Kazanjian S (2001). The reproducibility of organ position using active breathing control (ABC) during liver radiotherapy. Int J Radiat Oncol Biol Phys.

[7] Gagel B, Demirel C, Kientopf A (2007). Active breathing control (ABC): determination and reduction of breathing-induced organ motion in the chest. Int J Radiat Oncol Biol Phys.

[8] Panakis N, McNair HA, Christian JA (2008). Defining the margins in the radical radiotherapy of non-small cell lung cancer (NSCLC) with active breathing control (ABC) and the effect on physical lung parameters. Radiother Oncol.

[9] McNair HA, Brock J, Symonds-Tayler JR (2009). Feasibility of the use of the Active Breathing Co ordinator (ABC) in patients receiving radical radiotherapy for non-small cell lung cancer (NSCLC). Radiother Oncol.

[10] Lu L, Diaconu C, Djemil T, Videtic GMM, Abdel-Wahab M, Yu N (2018). Intra- and inter-fractional liver and lung tumor motions treated with SBRT under active breathing control. J Appl Clin Med Phys.

[11] Eccles CB, Brock K, Bissonnette J-P (2006). Reproducibility of liver position using active breathing coordinator for liver cancer radiotherapy. Int J Radiat Oncol Biol Phys.

[12] Keall PJ, Joshi S, Vedam SS, Siebers JV, Kini VR, Mohan R (2005). Four-dimensional radiotherapy planning for DMLC-based respiratory motion tracking. Med Phys.

[13] Shimohigashi Y, Toya R, Saito T, Ileda O (2017). Tumor motion changes in stereotactic body radiotherapy for liver tumors: an evaluation based on four-dimensional cone-beam computed tomography and fiducial markers. Radiat Oncol.

[14] Wunderink W, Mendez RA, Vasquez Osorio EM (2007). Target coverage in image-guided stereotactic body radiotherapy of liver tumors. Int J Radiat Oncol Biol Phys.

[15] Nakamura M, Shibuya K, Shiinoki T (2011). Positional reproducibility of pancreatic tumors under end-exhalation breath-hold conditions using a visual feedback technique. Int J Radiat Oncol Biol Phys.

[16] Court L, Rosen I, Mohan R, Dong L (2003). Evaluation of mechanical precision and alignment uncertainties for an integrated CT/LINAC system. Med Phys.

[17] Court LE, Dong L (2003). Automatic registration of the prostate for computed-tomography-guided radiotherapy. Med Phys.

[18] Zhang L, Dong L, Court L, Wang H, Gillin M, Mohan R (2005). Validation of CT-assisted targeting (CAT) software for soft tissue and bony target localization. Med Phys.

[19] Zhang L, Garden AS, Lo J (2006). Multiple regions-of-interest analysis of setup uncertainties for head-and-neck cancer radiotherapy. Int J Radiat Oncol Biol Phys.

[20] Wang H, Milgrom SA, Dabaja BS, Smith GL, Martel M, Pinnix CC (2017). Daily CT guidance improves target coverage during definitive radiation therapy for gastric MALT lymphoma. Pract Radiat Oncol.

[21] Remeijer P, Geerlof E, Ploeger L, Gihuijs K, van Herk M, Lebesque JV (2000). 3-D portal image analysis in clinical practice: an evaluation of 2-D and 3-D analysis techniques as applied to 30 prostate cancer patients. Int J Radiat Oncol Biol Phys.

[22] van Herk M (2004). Errors and margins in radiotherapy. Semin Radiat Oncol.

[23] Balter JM, Brock KK, Lam KK (2005). Evaluating the influence of setup uncertainties on treatment planning for focal liver tumors. Int J Radiat Oncol Biol Phys.

[24] Hawkins MA, Brock KK, Eccles C (2006). Assessment of residual error in liver position using kV cone-beam computed tomography for liver cancer high-precision radiation therapy. Int J Radiat Oncol Biol Phys.

[25] Case RB, Sonke JJ, Moseley DJ (2009). Inter- and intrafraction variability in liver position in non-breath-hold stereotactic body radiotherapy. Int J Radiat Oncol Biol Phys.

